# A New Spin on Photocatalysis: Vortex Fluidic‐Driven Photooxidations Enabled by Solution‐Processable Microporous Polymers

**DOI:** 10.1002/chem.71288

**Published:** 2026-07-09

**Authors:** Leonardo Amicosante, Fiona R. Gordon, Dominic Taylor, Neil B. McKeown, Daniel M. Jubb, Stuart R. Kennedy, Marc A. Little, Filipe Vilela, Colin L. Raston, Scott J. Dalgarno

**Affiliations:** ^1^ Institute of Chemical Sciences Heriot‐Watt University Edinburgh UK; ^2^ EaSTCHEM School of Chemistry University of Edinburgh Edinburgh UK; ^3^ The Falcon Project Limited Buckinghamshire UK; ^4^ Flinders Institute of Nanoscale Science and Technology, College of Science and Engineering Flinders University Adelaide SA Australia

**Keywords:** continuous flow, photocatalysis, porous polymers, singlet oxygen, vortex fluidics

## Abstract

Benzo[*c*][1,2,5]thiadiazole (BTZ) incorporated into polymers of intrinsic microporosity (PIM) has previously been observed as a highly effective and affordable approach for photocatalyzing both visible light‐driven energy transfer and single‐electron transfer reactions. In this work, newly developed PIM‐BTZ polymers (with commercially available monomers and facile synthesis) were applied as a reactive coating on the Vortex Fluidic Device (VFD), a rapid rotating tube reactor with continuous flow capabilities. The combination of the VFD's exceptionally high mass transfer properties and the photochemical properties of the PIM‐BTZ coating was shown to synergistically accelerate organic reactions involving singlet oxygen and superoxide species, while also enabling continuous flow operation and synthesis. Compared to batch mode, the VFD achieved a dramatic increase in productivity from 15.0 mg h^−1 ^to 126.4 mg h^−1^ when operated in continuous flow mode for the benchmark photooxidation of triphenylphosphine. Ultimately, the PIM‐BTZ coated VFD demonstrates robust capability as a scalable photochemical reactor.

## Graphical abstract

Newly developed polymers with intrinsic microporosity (PIMs) were combined with benzo[*c*][1,2,5]thiadiazole (BTZ) to be used as visible light photocatalytic coatings on the Vortex Fluidic Device (VFD). The VFD's rapid rotation and continuous flow capabilities enhanced singlet oxygen and superoxide radical reactions, achieving a dramatic eightfold increase in productivity compared to batch setups – demonstrating scalable photochemical efficiency.

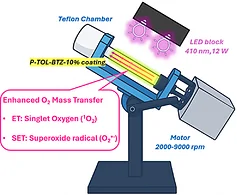

## 1 Introduction

In recent years, the increasing anthropogenic impact on the environment has heightened interest in developing alternative, greener chemical processes [1]. Among these, photocatalysis has garnered significant attention due to the potential environmental benefits of using light as a traceless reagent [2], with applications ranging from organic synthesis [3, 4, 5, 6] to solar fuel production [7, 8, 9, 10] and water treatment [11, 12, 13]. In particular, the direct utilisation of atmospheric oxygen as a versatile oxidant through photogenerated reactive oxygen species (ROS) is an enticing prospect. This reaction process includes the generation of species such as the superoxide radical anion (O_2_^•−^) via electron transfer from the photoredox catalyst (PRC) in its first excited electronic singlet (S_1_) state or from excited triplet (T_1_) state [5, 14, 15, 16], or singlet oxygen (^1^O_2_) by triplet‐triplet annihilation from the T_1_ of a photosensitizer [5, 17, 18, 19, 20].

While the efficacy and validity of photocatalysis, specifically of organic‐based PRC, have been proven extensively [21], arguably their greatest advantage lies in their structural versatility and adaptability, especially when based on electron donor‐acceptor (EDA) type structures, which are readily tuneable. Examples of EDA‐type structures that have gained recent traction in photocatalysis are those based on the benzo[*c*][1,2,5]thiadiazole (**BTZ**) electron acceptor unit. When used in conjunction with an electron donor, **BTZ** EDA‐type structures can exhibit strong light absorption along with good photostability and synthetic tunability (Figure 1) [22, 23, 24, 25, 26, 27]. The synthetic versatility of the **BTZ** group has seen it not only utilized as a molecular photocatalyst, but also incorporated into various materials such as linear or crosslinked polymers [28, 29], covalent organic frameworks (COFs) [30, 31], and metal‐organic frameworks (MOFs) for various light harvesting applications [4, 32].

**FIGURE 1:**
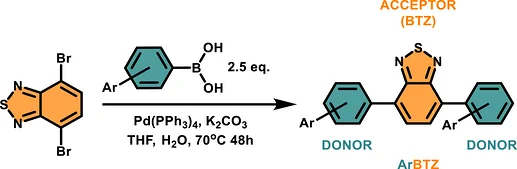
General structure of benzo[*c*][1,2,5]thiadiazole (**BTZ**) based PS and PRC, commonly synthesized via cross‐coupling strategies such as the Suzuki‐Miyaura reaction. Electron acceptor **BTZ** unit highlighted in orange and electron donor aromatic groups highlighted in teal.

Taylor et al. recently demonstrated that the incorporation of **BTZ** into polymers of intrinsic microporosity (PIMs) [33] resulted in photoactive materials as heterogeneous PS for use in fixed‐bed reactors [34]. The polymer backbones of PIMs are rigid and highly contorted, preventing the chains from close packing. As such, PIMs can display high degrees of microporosity arising from interconnected void spaces, in addition to excellent solubility arising from the disrupted interchain interactions [35]. By incorporating up to 5% of **BTZ** into the polymer **PIM‐EA‐TB**, which is a prominent PIM derived from the Tröger's base (TB) motif, the authors were able to dope the material with 5% **BTZ** (**PIM‐BTZ‐5%**) to impart photosensitizing properties without compromising the porosity (measured surface area of 914 m^2^ g^−1^) or solubility compared to the pristine polymer. By taking advantage of the inherent solubility of the modified PIM in chloroform, **PIM‐BTZ‐5%** was coated onto glass beads as an optically inert support. These beads were then packed into a glass column for continuous flow photooxidation of triphenylphosphine via ^1^O_2_ (Figure 2A).

**FIGURE 2:**
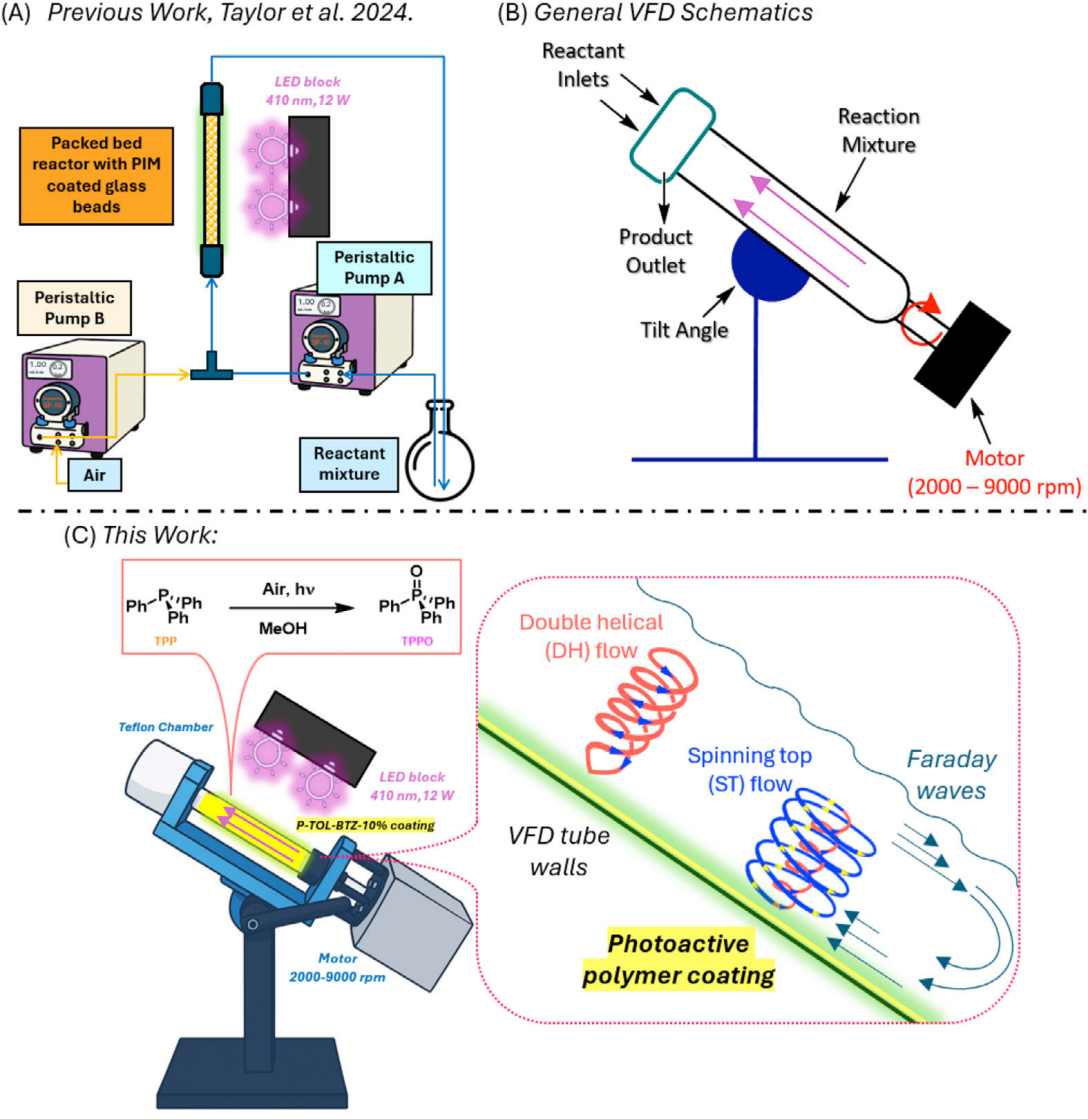
(A) Previous work by Taylor et al. was concerned with the immobilization of photoactive **BTZ** PIM on glass beads for use in heterogeneous photooxidation in flow [34]. (B) Labelled schematic of the Vortex fluidic device (VFD). (C) Present work, VFD coated in **BTZ** PIM heterogeneous photocatalyst material, with description of fluid flow topologies generated via the VFD's high rotation speed capabilities.

Recent interest in sustainable chemistry has also focused on technological innovations to extract greater efficiency from reactions. Spinning reactors, including the Taylor Vortex Reactor (TVR), Spinning Disc Reactor (SDR) and the Vortex Fluidic Device (VFD), have been observed to drive reactions more effectively through intense micromixing and high mass and heat transfer [36]. As further discussed in a review by Soria‐Castro et al., these reactors have been involved in photooxidations with Rose Bengal for the TVR, pollutant removal with titanium dioxide for the SDR, and graphene oxide reduction aiding material transformations for the VFD [36]. In this current study, the authors have further explored the capabilities of the VFD: a microfluidic reactor consisting of a quartz sample tube that can typically be rotated at speeds up to 9000 rpm and tilted at different angles (Figure 2B). Once the reaction mixture is added to the tube, centrifugal forces push the liquid up the tube and around the inside walls to generate thin films with induced high shear mechanical energy, which can be harnessed to accelerate reactions [37]. A tilt angle of 45° has been previously reported to be optimal for applications of the thin film microfluidic platform [38]. The VFD can be used in the confined mode by closing the open top end of the tube containing a finite volume of liquid. Alternatively, the VFD can be operated in continuous flow mode, allowing scalability by constantly delivering liquid/reagents to the base or desired positions along the tube, which then exit at the top of the system.

The VFD has been used in a range of applications, including the development of organic shampoo formulation [39, 40], processability of carbon nanotubes [41, 42, 43], and reducing the reaction times for many small molecule transformations [44, 45]. The chemical enhancement of reactions in the VFD arises from the intense micromixing and high shear stress within well‐defined topological fluid flows of micron to submicron in diameter, where there is high heat and mass transfer, with high localised pressures, in excess of 20 MPa [46]. Spinning top (ST), typhoon‐like, flow from the Coriolis force is generated at the base of the tube, which then moves up the tube, along with Faraday waves generated along the length of the tube, where the associated eddies are twisted into double helical (DH) flow by side wall Coriolis forces (Figure 2C) [39, 41, 47, 48, 49]. The latter, in confined mode, are more prevalent at higher rotational speeds (> 6000 rpm), with their diameters decreasing in a step fashion as the rotational speed increases. In contrast, the ST flow is more prevalent at lower speeds (< 5000 rpm), with its diameters increasing concomitantly with rotational speed [47]. As the diameter of the ST increases, its mechanical energy is spread over a larger volume, and this is consistent with the impact of VFD processing diminishing above 7,500 rpm [36, 47]. In addition, previous studies concluded that as the speed increases, the film thickness (uniform along the length of the tube) decreases stepwise. This is consistent with the presence of Faraday waves associated with DH flow, and across each step (in rpm) for increasing rotational speed, while the product of the rotational speed and the pitch of the double helix is constant. Thus, at a critical speed, the DH is disrupted, and the film spontaneously becomes thinner, creating a new step at the slightly higher rotational speed [47].

Photocatalytic reactions in particular benefit from irradiation of the tube, where the thin film of liquid tracks toward a uniform exposure of the reacting species, which is thus favoured at higher rotational speeds, where centrifugally the film of liquid will be thinner than at lower rotational speeds [50]. The formation of thin films of reagents is also particularly beneficial to photocatalysis due to the more effective irradiation as a consequence of the reduction in optical path length of solution described by the Beer−Lambert law. Continuous flow processing potentially disturbs the formation of Faraday waves and associated double helical flow, with the dominant topological flow now being from the Coriolis at the base of the tube, as spinning top flow. Overall, the VFD is an ideal platform for using field effects to drive processing in the device, the most recent being for magnetic fields [46].

In this study, a series of three solution‐processable PIMs containing 10 mol% of **BTZ** as the light‐harvesting component were prepared and then used to coat the interior walls of the VFD tube (Figure 2C). The PIM‐coated VFD tubes were then tested for the production of ^1^O_2_ and O_2_^•−^, using benchmark reactions including the oxidation of triphenylphosphine and hydroxylation of boronic acid, in both confined and continuous flow modes of operation. Continuous flow processing using the **BTZ**‐doped PIM‐coated VFD tube was used to exploit the VFD's high mass transfer abilities and enhance scalability and productivity.

## 2 Results and Discussion

### 2.1 Synthesis and Characterisation of BTZ‐Doped Polymer Materials

TB porous polymers are usually prepared by the in situ formation of the rigid, bicyclic TB linkage between amine groups using dimethoxymethane (DMM) and trifluoroacetic acid (TFA) as the catalyst [35]. Judicious selection of the amine groups allows the porosity and solubility of the resultant porous polymer to be controlled. This approach has been successfully utilized by Carta et al. to generate solution‐processable polymers for gas separation membranes, such as the porous polymer **PIM‐EA‐TB** with high apparent surface area [51]. More recently, Taylor et al. successfully incorporated a diamine‐modified **BTZ** into **PIM‐EA‐TB** in order to generate a photoactive polymer with inherent microporosity and solubility [34]. However, this approach is limited by the use of ethanoanthracene (**EA**) as a bespoke polymer building block, as it requires a multistep synthesis. A central aspect of the work presented here was to make use of commercially available aromatic diamines as monomers for copolymerisation with diamino‐functionalized **BTZ** to generate **BTZ**‐doped TB polymers (Figure 3).

**FIGURE 3:**
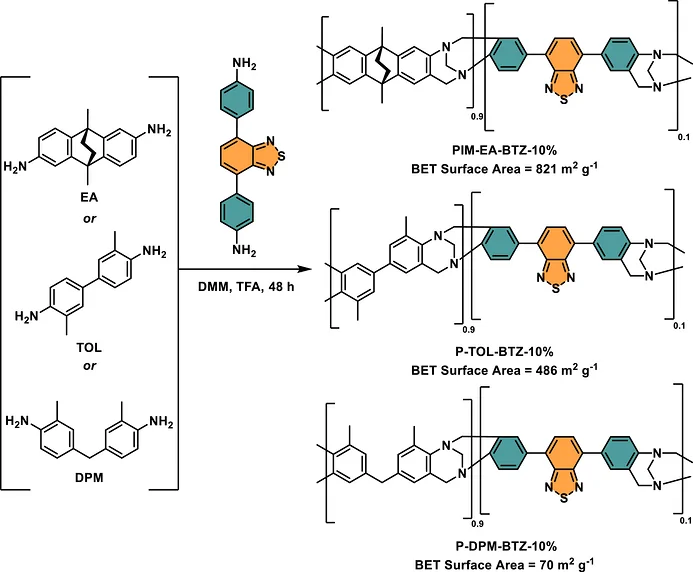
Preparation of **BTZ**‐doped PIMs by Tröger's base formation.

With this goal in mind, incorporation of **BTZ** into TB polymers based on commercially available tolidine (**TOL**) and di(aminophenyl)methane (**DPM**) monomers was the initial objective. These novel TB polymers were prepared using a procedure initially developed by Carta et al., and later adapted by Taylor et al. for the incorporation of **BTZ** groups, as yellow powders [34, 35]. The PIMs are referred to as **P‐TOL‐BTZ‐10%** and **P‐DPM‐BTZ‐10%** to describe the 10 mol% of **BTZ** present in the feed ratio for the polymerisation and the **TOL**/**DPM** comonomers. To provide suitable comparisons for the effect of the **BTZ** groups on the properties of the PIMs, the undoped analogues **P‐TOL‐TB** and **P‐DPM‐TB** were also prepared by the same method, which yielded two white powdered samples. A 10% **BTZ**‐doped analogue of **PIM‐BTZ‐5%**, designated **PIM‐EA‐BTZ‐10%**, was also prepared. The solubility of the polymers in CDCl_3_ allowed for thorough characterisation by ^1^H and ^13^C NMR spectroscopy. The ^1^H NMR spectra of **PIM‐EA‐BTZ‐10%**, **P‐TOL‐BTZ‐10%**, and **P‐DPM‐BTZ‐10%** were compared with the spectra of the undoped **PIM‐EA‐TB**, **P‐TOL‐TB**, and **P‐DPM‐TB** (Figures S3–S12). The presence of additional peaks in the region of 7.00 – 8.00 ppm in the spectra of the **BTZ**‐doped polymers indicated successful incorporation of **BTZ**, and by comparing the ^1^H NMR spectra of the **BTZ**‐doped and nondoped TB polymers, it was possible to estimate the actual **BTZ** content as 9.9% for **PIM‐EA‐BTZ‐10%**, 11.1% for **P‐TOL‐BTZ‐10%,** and 10.1% for **P‐DPM‐BTZ‐10%**. The presence of **BTZ** groups was also confirmed by performing UV‐Vis absorption spectroscopy in chloroform solution: while **PIM‐EA‐TB**, **P‐TOL‐TB** and **P‐DPM‐TB** did not exhibit any visible light absorption, all three 10% **BTZ**‐doped polymers exhibited a well‐defined absorption peak with a maximum at 413 nm (Figures S13–S15). This new absorption peak arises as a result of intramolecular charge transfer (ICT) from the phenyl groups/TB linkages to the **BTZ** groups: this ICT band is a common feature of **BTZ**‐based electron donor‐acceptor systems [52].

To assess the porosity and surface area of the **BTZ**‐doped TB polymers, gas adsorption/desorption analysis was conducted using N_2_ at 77 K and CO_2_ at 273 K as the adsorbates. The N_2_ adsorption/desorption isotherms for all three **BTZ**‐doped polymers show Type IV shapes, indicative of materials with both micro‐ and mesoporous features (Figures S17–S19) [53]. The highest Brunauer‐Emmett‐Teller (BET) surface area of 821 m^2^ g^−1^ was measured for **PIM‐EA‐BTZ‐10%**, which is a drop from the reported surface area of 1035 m^2^ g^−1^ for **PIM‐EA‐TB** [51]. This was, however, higher than the BET surface areas measured for **P‐TOL‐BTZ‐10%** or **P‐DPM‐BTZ‐10%**, calculated as 486 and 70 m^2^ g^−1^, respectively. These values compared relatively well to the BET surface areas of the undoped TB polymers of 502 and 26 m^2^ g^−1^ for **P‐TOL‐TB** and **P‐DPM‐TB**, respectively. The decreasing trend of **EA** > **TOL** > **DPM** in the BET surface area reflects the decrease in the structural rigidity of the monomer building blocks, allowing the polymer chains to pack more closely, thus eliminating intrinsic microporosity. Therefore, the **TOL** group represents a useful intermediate between the synthetically challenging **EA** group and the nonporous **DPM** group. CO_2_ gas adsorption isotherms recorded at 273 K show uptake of 62, 38, and 21 cm^3^ g^−1^ at 1 bar of pressure for **PIM‐EA‐BTZ‐10%**, **P‐TOL‐BTZ‐10%** and **P‐DPM‐BTZ‐10%**, respectively (Figure S20). As was the case for the N_2_ adsorption/desorption isotherms, these CO_2_ adsorption values are similar to the undoped polymers **P‐TOL‐TB** and **P‐DPM‐TB** (43 and 20 cm^3^ g^−1^ at 1 bar, respectively), highlighting that **BTZ** incorporation into the polymers does not significantly reduce the porosity of the material. The pore size distribution of each of the polymers was calculated using nonlocal density functional theory (NLDFT), which revealed that all of the materials had significant micropore density in the range of 0.4–0.7 nm, following the same **EA** > **TOL** > **DPM** trend (Figures S21–S25).

### 2.2 Forming the Photosensitizer Coating

The formation of the photosensitizer coating on the walls of the quartz VFD tube was investigated using different methods. First, the PIM photosensitizers were dissolved in a minimum volume (2 mg of material in 0.4 mL of solvent) of dichloromethane (DCM) or chloroform to form coating solutions that, upon evaporation of the solvent, enabled a film to be cast on a surface. Several evaporation methods were tested, including using a heat gun (low setting for gentle heating) to encourage evaporation of the solvent in the VFD tube while rotating horizontally at 500 rpm (as had previously proven to be effective) [37]. However, this resulted in a striped, patchy coating (Figures S26–S28). A rotary evaporator was also used to slowly remove the solvent while manually changing the angle to more evenly distribute the coating. However, this approach also did not yield the desired uniform coating (Figure S29). The optimal uniform coating was formed using the VFD itself. Using a long needle and syringe, a concentrated solution of the PIM photosensitizer dissolved in DCM was slowly injected into the VFD tube initially at its base, then up the tube while it was rotated at 6000 rpm at a 45° angle. The resulting coating appeared much more uniform than previous methods (Figure 4). The durability of this coating was tested by rotating deionized water and methanol for 24 h at 6000 rpm. These solvents were chosen as they do not dissolve the PIM coating, unlike chloroform and DCM. Solvent compatibilities of PIM‐**BTZ** photosensitizers have been previously discussed by Taylor et al. [34]. Deionized water was shown to be a more viscous liquid than methanol upon spinning, resulting in trace amounts of delamination compared to methanol, which showed no delamination and a fully intact coating after 24 h rotating (Table S2).

**FIGURE 4:**
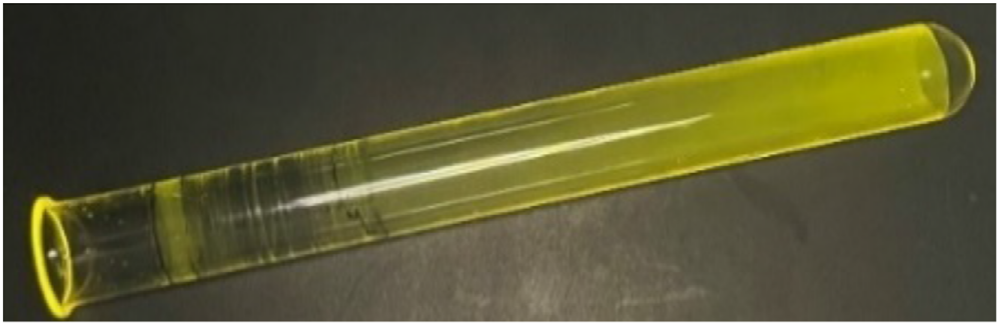
Uniform **P‐TOL‐BTZ‐10%** photosensitizer coating formed via the VFD high rotation injection method. The VFD glass quartz tube has dimensions of 20 mm outer diameter (OD) x 17 mm inner diameter (ID) x 190 mm length.

### 2.3 Oxidation of Triphenylphosphine

The oxidation of triphenylphosphine (TPP) to triphenylphosphine oxide (TPPO) is an excellent benchmark reaction for photosensitizers as it proceeds via the production of ^1^O_2_ in aerobic conditions [34]. Targeting this reaction, initial investigation using the **P‐TOL‐BTZ‐10%** coated tube in the VFD in confined mode demonstrated promising results with 82% conversion after 30 min (Table 1, Entry 1). The percentage conversion was calculated using quantitative ^31^P NMR experiments (see Supporting Information) verified by use of an internal standard (diphenylphosphine oxide). Control experiments were carried out to confirm that each component of the experiment was required, e.g., photosensitizer, rotation, and LED light (Table 1). The wavelength of the LED light was chosen to be 410 nm, as it corresponds to the absorption wavelength from the UV‐Vis spectrum of the **BTZ** polymers to be investigated (Figure S13–S15). The use of the VFD rotation at 2000 rpm showed significantly larger conversion compared to a control experiment where the tube was not spun (Table 1, Entry 1 vs Entry 3). The reaction was also observed to proceed slowly with only 410 nm LED light in the absence of both photosensitizer and rotation. Overall, it was confirmed that each parameter was required to achieve high conversions in 30‐minutes.

**TABLE 1:** Control experiments for the oxidation of triphenylphosphine using the VFD in confined mode. For each experiment, a 45° tilt angle was used along with 0.05 mmol of triphenylphosphine. Each experiment was carried out for 30 minutes.

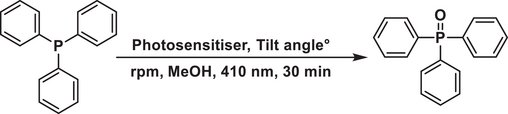				
Entry	P‐TOL‐BTZ‐10%	Rotation Speed/rpm	410 nm light	Conversion / %^a^
1	+	2000	+	**82**
2	+	2000	−	**0**
3	+	0	+	**38**
4	−	2000	+	**6**
5	−	2000	−	**0**
6	−	0	+	**11**
7	−	0	−	**0**

^a^ Percentage conversion calculated using reactant and product integrals from quantitative ^31^P NMR experiments.

The reusability and reproducibility of the photosensitizer coating were subsequently investigated. The thickness of the coating consisted of 2 mg covering a surface area of 1816 cm^2^. Using the literature density for PIM materials, the coating could be estimated to be 110 nm thick, thus within the range of previously reported literature values (180–80 nm) [54, 55] Overall, polymer thickness was established to be of secondary impact to reactivity compared to pore size, as determined by the low difference in reactivity tests at higher PIM concentration (please see Supporting Information). The polymer coating was reused over five consecutive experiments, yielding an average conversion of 86% (± 4.3%) (Figures S31, S32) for the oxidation of triphenylphosphine (0.05 mmol) in methanol at 2000 rpm with a tilt angle of 45°. Optical transmittance was analysed using an optical power/energy meter (Thorlabs PM100D, Figure S30 and Table S3) to determine the coverage and consistency and confirm that only slight delamination of the coating occurred after each of these experiments. The heterogeneous photosensitizer PIM coating was also required to be reliably reproduced by other users. Relative consistency over three reproduced coatings was determined via optical transmittance (Table S4). The reproduced coatings for the oxidation of triphenylphosphine gave similar conversions with an average of 80% (± 2%) (Figures S33, S34), confirming that the coating could be reliably reproduced.

The oxidation of triphenylphosphine was then optimised to find the parameters to give the highest conversion after 30 min (Table 2). The VFD's tilt angle can be altered between 0° (horizontal) and 90° (vertical). The 45° tilt angle was found to give the best reaction conversions as it provides a favourable gradient to allow the centrifugal forces to efficiently push the reaction mixture up and around the tube to form a thin film solution to make contact against the **P‐TOL‐BTZ‐10%** coating [38, 44]. The second optimisation parameter investigated was the rotation speed. Jellicoe et al. described how, at different rotation speeds, different forces impact the way the fluid moves in the VFD tube [37]. The centrifugal forces pushing the reaction mixture up the coated walls of the tube are driven by the VFD's high rotation speeds. Optimum rotation promotes high contact between the reaction mixture thin films, the coated **P‐TOL‐BTZ‐10%** tube walls, and the gas phase. Experiments at a 45° tilt angle with the **P‐TOL‐BTZ‐10%** coated tube at 6000 and 7500 rpm yielded the optimum conversion results after 30‐minutes, reaching up to 94% (Table 2, Entries 9 and 10). Using these optimal VFD parameters of angle (45°) and rotation speed (6000 rpm), the different **BTZ** photosensitizing polymer materials and their blank polymers were also investigated. It was observed that with equal loading of photosensitizer **BTZ** unit (scaled based on molecular weight of repeating units), the polymer with the highest performance was **P‐TOL‐BTZ‐10%**, which at optimal conditions reliably obtained 94% conversion in 30 minutes (Table 2, Entry 9). The resulting measured trend in reactivity was **P‐TOL‐BTZ‐10%** > **PIM‐EA‐BTZ‐10%** > **P‐DPM‐BTZ‐10%**. The tests with the blank polymers, **P‐TOL‐TB**, **PIM‐EA‐TB**, and **P‐DPM‐TB**, gave different results compared to the control experiment with no photosensitizer present. A possible inference was that the polymer alone plays no photosensitizer role but rather accommodates the substrate and presents a greater volume of oxygen in its pores in VFD tests. Overall, the optimum results were obtained at 6000 rpm with a 45° tilt angle in the **P‐TOL‐BTZ‐10%** coated VFD tube. The highest conversions of 94% were achieved after a 30‐minute reaction time for the oxidation of triphenylphosphine, and complete conversion was observed by the 40‐minute mark (Table 2, Entry 17). Previous investigations by the group into **PIM‐BTZ‐5%** coated glass beads in a packed bed column reactor in flow gave 90% conversion after 18 h in methanol [34], however, a more appropriate comparison is given by considering the space‐time yields (*vide infra*).

**TABLE 2:** Optimization of the oxidation of TPP into TPPO in the VFD via ^1^O_2_.

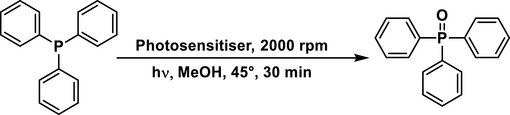					
Entry	Photosensitizer	Tilt angle / °	Rotation speed/rpm	Time/mins	Conversion/%^a^
1	**P‐TOL‐BTZ‐10%**	0 (horizontal)	2000	30	**80**
2	**P‐TOL‐BTZ‐10%**	30	2000	30	**82**
3	**P‐TOL‐BTZ‐10%**	45	2000	30	**85**
4	**P‐TOL‐BTZ‐10%**	60	2000	30	**77**
5	**P‐TOL‐BTZ‐10%**	90 (vertical)	2000	30	**58**
6	**P‐TOL‐BTZ‐10%**	45	3000	30	**84**
7	**P‐TOL‐BTZ‐10%**	45	4500	30	**81**
8	**P‐TOL‐BTZ‐10%**	45	5250	30	**89**
9	**P‐TOL‐BTZ‐10%**	45	6000	30	**94**
10	**P‐TOL‐BTZ‐10%**	45	7500	30	**93**
11	**P‐TOL‐BTZ‐10%**	45	9000	30	**91**
12	**P‐TOL‐TB**	45	6000	30	**21**
13	**PIM‐EA‐BTZ‐10%**	45	6000	30	**64**
14	**PIM‐EA‐TB**	45	6000	30	**15**
15	**P‐DPM‐BTZ‐10%**	45	6000	30	**52**
16	**P‐DPM‐TB**	45	6000	30	**6**
17	**P‐TOL‐BTZ‐10%**	45	6000	40	**100**

^a^ Percentage conversion calculated using reactant and product integrals from quantitative ^31^P NMR experiment.

An advantage of the VFD photosensitizer‐coated tube is the higher amount of ^1^O_2_ produced compared to batch modes in a vial with no stirring or rotation. This was observed using DABCO, a known ^1^O_2_ quencher (Table S4) [56]. Typically, only 2–10 molar equivalents of DABCO are necessary to stop the reaction altogether [56], as revealed in batch, in a vial, where 10 molar equivalents of DABCO resulted in no reaction progression. The same could be observed when the **P‐TOL‐BTZ‐10%** photosensitizer was not coated, but rather free in the reaction mixture of triphenylphosphine in methanol in the VFD at 45° tilt angle and rotated at 2000 rpm. However, when the material was coated on the VFD tube and spun at the same conditions, 10 molar equivalents of DABCO resulted in 50% conversion and was thus unable to stop the reaction. This was still lower than the average 85% conversion observed after a 30‐minute reaction with these experimental parameters, implying that DABCO was quenching at least some of the ^1^O_2_, preventing it from reacting with the substrate. Increasing the molar equivalents to 26 still showed some progression with 18% conversion after 30 min, and a further increase to 64 molar equivalents of DABCO finally prevented the oxidation from proceeding, resulting in no conversion of TPP to TPPO. This investigation highlighted the effectiveness of the photosensitizer material coated onto the VFD tube surface, as it resulted in higher ^1^O_2_ production (quenched only by large amounts of DABCO). This may be attributable to efficient oxygen gas exchange and greater exposure of the reacting solution to the photoactive coating because of the thin film formed by the VFD. Therefore, the VFD was deemed responsible for obtaining an increased surface area to oxygen volume ratio compared to other reactors.

### 2.4 Homogeneous Photosensitizer Oxidation of Triphenylphosphine Oxide

Comparison was made between using **P‐TOL‐BTZ‐10%** as a heterogeneous and homogeneous photosensitizer (Table S7). Experimental conditions for the homogeneous photosensitizer include using chloroform as the reaction solvent, as the PIM readily dissolves in this solvent. However, the use of chloroform detrimental to green chemistry principles due to its hazardous properties as discussed in the CHEM21 solvent selection guide [57]. In the homogeneous reaction, 2 mL of chloroform is used for each experiment in contrast to 0.4 mL of chloroform or DCM to coat the heterogeneous photosensitizer, which endures at least five consecutive experiments. The homogeneous reaction in chloroform does lead to 100% completion after a shorter reaction time of 10 min in confined mode, however, due to not being able to recover the photosensitizer and using a larger volume of problematic solvents, the heterogeneous method is favoured [1] (Scheme 1 and 2).

**SCHEME 1:**
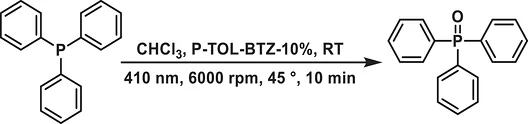
Reaction scheme for the oxidation of triphenyl phosphine using homogeneous photosensitizer conditions.

### 2.5 Oxidation of 2‐furoic Acid

In order to widen the scope of uses for the photosensitizing film, other reactions (both based on ^1^O_2_ and other ROS) were explored, utilising the polymer materials described above. The first reaction chosen was the oxidation of 2‐furoic acid, which also occurs via ^1^O_2_ [18].

**SCHEME 2:**
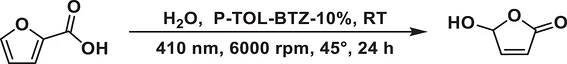
Reaction scheme of the oxidation of 2‐furoic acid via ^1^O_2_ mechanism in deionized water.

The reagent is soluble in water, which has an even shorter ^1^O_2_ lifetime of approximately 3.1 µs compared to 9.1 µs in methanol [58]. The inherently poor lifetime of ^1^O_2_ in the reaction media results in the reaction time to reach completion being substantially longer. Taylor et al. previously reported only 30% conversion for the oxidation of 2‐furoic acid using **PIM‐BTZ‐5%** coated glass beads in flow conditions [34]. The VFD tests with the **P‐TOL‐BTZ‐10%** coated tube in deionised water showed outstanding performance, reaching full completion after only 24 h. This again emphasises the high oxygen mass transfer of the VFD along with the enhanced production of ^1^O_2_ by the photosensitizing **BTZ**‐doped PIM coating, leading to a significant improvement in conversion that mitigates the need for water as a solvent. Moreover, as shearing and degradation of the polymer coatings in water at 2000 rpm were minimal, this system is deemed suitable for water treatment applications, given the high production of ^1^O_2_ in water and the traceless nature of the heterogeneous photosensitizer material.

### 2.6 Hydroxylation of 4‐(Trifluoromethyl)Phenyl Boronic Acid via Superoxide Radical

**BTZ**‐doped materials can take part in single electron transfer reactions, acting as photoredox catalysts instead of photosensitizers. A useful test reaction for such a mechanism comprises the generation of the O_2_^•−^ radical, which can be utilized in organic transformations such as the hydroxylation of aryl‐boronic acids. This reaction still requires aerobic conditions and was thus deemed useful to be applied in the VFD system as it can still benefit from the higher oxygen mass transfer benefits discussed above. In addition, this reaction also requires a sacrificial electron donor to complete the photoredox cycle (in this case, diisopropylethylamine) [59, 60]. Control experiments, with reaction conversions calculated using ^19^F NMR (Figures S35, S36) measurements, were conducted over 2 h to show that each feature of the reaction was required (Table 3). It was observed that some conversion was seen when no reductant was used. This effect was also noted in the literature, where trace amounts of product are synthesized in the absence of the sacrificial reductant during control tests [59, 60], and while in batch, this effect converts only a small amount of boronic acid over a long period, the tests in the VFD yielded 19% conversion in 2 h without a sacrificial electron donor. Radical trapping and scavenger experiments were also carried out as part of the control tests, concluding that both DABCO and TEMPO were required to completely halt the reaction (Table S8). The VFD was proven above to accelerate the rate of reaction by providing higher mass transfer of oxygen reagent while spinning at 6000 rpm with photocatalyst coating. Therefore, it was hypothesized that this reactivity in the absence of reductant was also dependent on the availability of oxygen and hence increased due to the VFD system, since tests with the **BTZ**‐doped PIM materials in batch gave trace conversions.

**TABLE 3:** Control experiments for the hydroxylation of 4‐(trifluoromethyl)phenyl boronic acid using the VFD in confined mode. For each experiment, a 45° tilt angle was used along with 0.05 mmol of 4‐(trifluoromethyl)phenyl boronic acid. Each experiment was carried out for 2 h. Conversions calculated using ^19^F NMR.

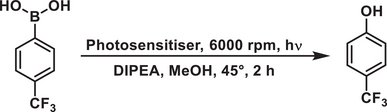					
Entry	P‐TOL‐BTZ‐10%	Rotation speed/rpm	DIPEA	410 nm light	Conversion/%^a^
1	+	6000	+	+	**55**
2	+	6000	+	—	**0**
3	+	6000	+	+	**19**
4	+	—	+	+	**19**
5	—	6000	—	+	**3**
6	—	6000	+	—	**2**
7	—	6000	—	—	**0**

^a^ Percentage conversion calculated using reactant and product integrals from ^19^F NMR experiment.

Optimization of this reaction in the VFD was performed over 2 h and showed similar trends to the triphenylphosphine optimization. Overall, it was found that a rotation speed of 7500 rpm at 45° tilt angle with the **P‐TOL‐BTZ‐10%** PRC coating gave the best results over 2 h with 63% conversion (Table S8), and full conversion in 4 h. This can be compared to previous studies by Tobin et al. using heterogeneous styrene‐**BTZ** gels observed the hydroxylation to gain full conversion over 18 h under batch conditions [5].

### 2.7 VFD in Continuous Flow Mode

Beyond studying the VFD's ability to enhance photooxidation reactions in confined mode through high oxygen mass transfer and intense mixing, the fundamental limitation of the VFD reactor to address was scalability. Scalability of photoredox‐catalyzed reactions is nontrivial as they are driven by absorption of photons and hence their behaviour is governed by the Beer‐Lambert law. This relates the absorbance (A) of a solution to its molar attenuation coefficient (ɛ), molar concentration (c), and the optical path length (l) in cm as described in equation 1.

(1) A=εcl

A consequence of the Beer−Lambert law is that scaling up a photoreaction in batch leads to a significant variation in absorption experienced across the reaction vessel, which leads to inconsistent irradiation [61]. The same problem occurs with directly scaling up the size of the VFD system, albeit to a lower extent, due to the thin film formation around the walls during the spin. Therefore, while confined mode showed outstanding performance toward the tested photooxidation reactions, the tube volume limits the scale to mmol of material, which needs to be loaded and recovered manually by an operator. To effectively use the VFD, no more than 2 mL reaction mixture should be used, as this allows for thin films to form against the walls of the tube, generating shear stress to accelerate the reaction. The VFD was employed in continuous flow to tackle these limitations. To do so, the VFD tube was connected to a flask containing the reagents, which was carried to the reactor by a pump (pump A), injecting the reaction mixture into the tube from the bottom with the aid of a long needle (Figure 5). The tube was coated with photoactive **P‐TOL‐BTZ‐10%**, with the top end open to allow the reaction mixture to be added while enabling a larger volume of oxygen to enter. As the reaction mixture was carried to the bottom of the tube, centrifugal forces pushed it up and over the coated walls of the tube, increasing contact with the **P‐TOL‐BTZ‐10%** coating. The mixture eventually reached the top of the tube, entering a Teflon lid chamber, where they were carried out of the VFD by another pump (pump B, 5 mL min^−1^) via tubing into another flask to collect product (Figure S2). Optimisation of the oxidation of triphenylphosphine using this mode observed full conversion after a residence time of 18‐minutes with pump A set at a flow rate of 0.1 mL/min (Table S10). Pump B was set at a higher rate of 5 mL/min to ensure that the Teflon collection chamber was emptied as quickly as possible, minimising potential liquid leakage.

**FIGURE 5:**
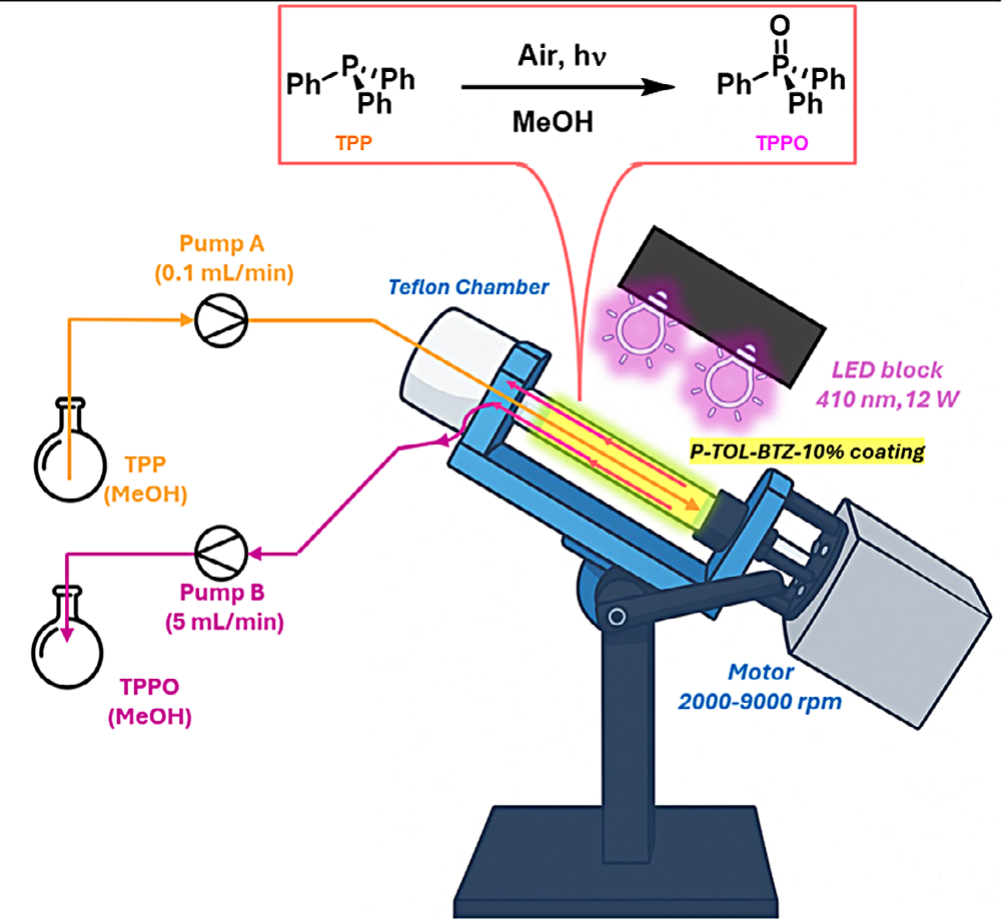
Schematic of the VFD in continuous flow mode for photooxidation of TPP into TPPO.

When compared to confined mode, which achieved full conversion in 40 minutes under optimised conditions, continuous flow mode showed promising enhancements to reactivity, which can be attributed to two major improvements that emphasise the effectiveness of the VFD system. The first was given by the replacement of the sealed test‐tube lid with the open Teflon chamber. This led to the amount of oxygen entering the reactor tube to be greater and thus enhanced reactivity by facilitating further oxygen mass transfer. The second was that a relatively low flow rate of 0.3 mL min^−1^ for pump A meant that each drop of reaction mixture was spun to the walls of the tube, encountering a greater loading compared to the confined mode, where the whole reaction mixture volume is spun at the same time on the same surface. Each unit volume of reactant therefore experiences a relatively higher amount of oxygen and photoactive materials (and pores) since the ratio of active surface area vs. volume of reaction increased. Additionally, increasing the flow rate was observed to decrease the conversion (Table S10), which is likely due to the reduced length of time each drop of reaction mixture is exposed to the excited photosensitizer coating. The tube does not completely fill up with reaction mixture, and the sample tube contained approximately 1.8 mL before the solution leaves the reactor and is collected and analysed. The reaction parameters can be entered into a space‐time yield calculation to compare production with batch and literature. The space‐time yield for the oxidation of triphenylphosphine in the VFD was calculated as 23 mg h^−1^ mL^−1^, thus almost double with respect to the confined mode (13 mg h^−1^ mL^−1^) and over threefold greater than batch (7.5 mg h^−1^ mL^−1^) (see Table S11, equations S2–S8).

### 2.8 3D‐Printed Photoreactor Shield

For all abovementioned tests, while the reaction angles, conditions, and mode of the VFD were altered and explored, the photoreactor setup was kept fundamentally identical. In this setup, a 410 nm LED block was attached to the surface of a transparent plastic protective shield of the VFD with tape, and the rest of the surface was covered with aluminium foil to block harmful light egress, but also to reflect it toward the reactor tube (Figures S1, S2). Whilst this was a cost‐effective method to obtain valid and reproducible data on the photoactivity in the VFD tube, the necessity for a more elegant design arose from the way the LED lamp and its heat sink were attached to the VFD shield. Therefore, a new photoreactor of the size of the old shield was designed using 3D modelling software (files available in Supporting Information) such that it could also function as a rest for the lamp. The resulting shield (Figures S37, S38) with reflective mirrors, granting internal reflectivity, was used in both confined mode and continuous flow, giving a small increase in conversion with 96% after 30 min in the latter, achieving 100% conversion after a much shorter 6 min residence time (Figure 6, Table S11). Thus, the space‐time yield also increased sharply to 70 mg h^−1^ mL^−1^.

**FIGURE 6:**
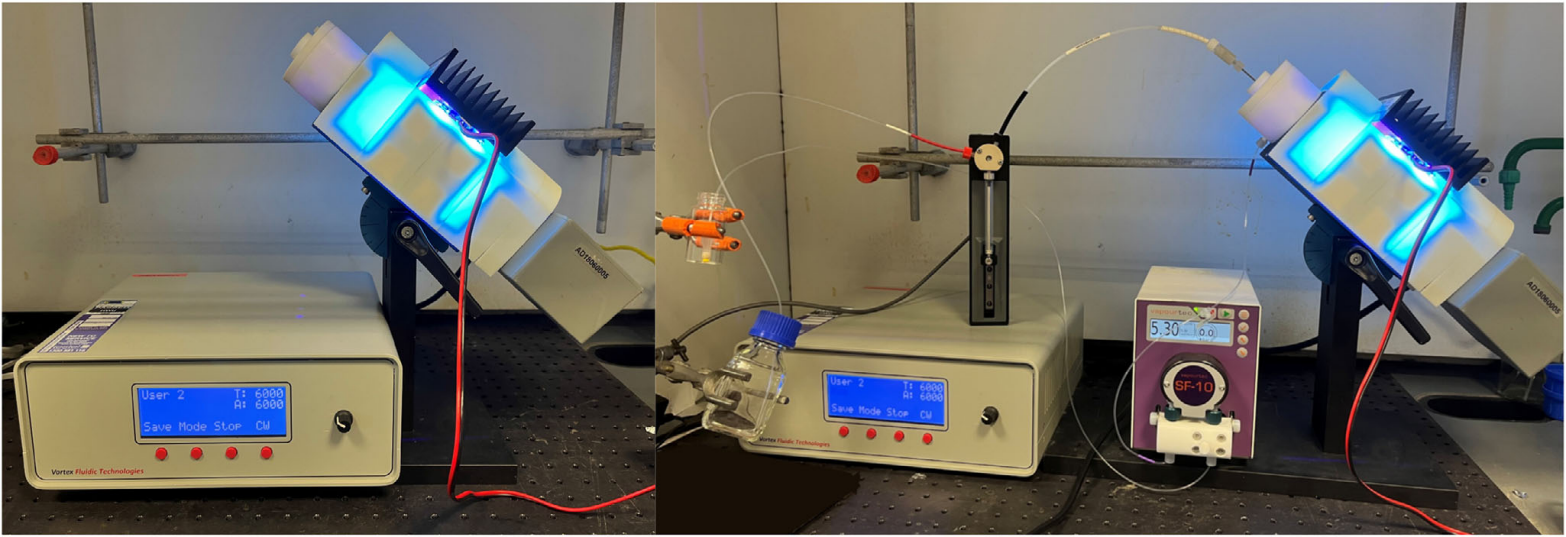
Picture of operating 3D printed photoreactor shield for VFD, with LED lamp rest, in both confined mode (left) and continuous flow mode (right).

## 3 Conclusion

Overall, the use of **BTZ**‐based PIM heterogeneous photocatalysts in a VFD reactor demonstrated significant advantages for continuous photochemical synthesis, improving reactivity, throughput, reusability, and efficiency. The **BTZ**‐doped PIM materials were readily synthesized and combined microporosity (ranging from 70–821 m^2^ g^−1^) with solubility (and therefore processability). VFD reactor tubes coated with these photochemical materials achieved higher conversions in benchmark photooxidation reactions, which we attribute to accelerated mass transfer of oxygen to the reaction mixture and photocatalyst, reducing the reaction time significantly and providing validation for this approach. The coated VFD reactors were tested for ^1^O_2_ production via the oxidation of TPP (in methanol), furoic acid (in water), and also O_2_^•−^ production in the hydroxylation of aryl‐boronic acids. These experiments demonstrated that the **BTZ**‐doped PIM‐coated VFD tubes successfully enhanced both ^1^O_2_ and O_2_^•−^ radical‐promoted photooxidations. To overcome the limited volume that can be used in confined VFD mode (2 mL), the reactor was used in conjunction with continuous flow chemistry, where the system was connected to two pumps, switching to continuous flow mode. We envisage that future studies will examine the use of automation to achieve rapid reaction optimisation and higher product throughput.

## Conflicts of Interest

The authors declare no conflicts of interest.

## Supporting information




**Supporting File**: The supporting information document contains all synthetic procedures, characterization of new materials and complete outline of the tests and VFD optimization experiments. The authors have cited additional references within the Supporting Information [4, 19, 33, 35, 58, 59].
chem71288‐sup‐0001‐SuppMat.docx

## Data Availability

The data that supports the findings of this study are available in the Supporting Information of this article.
