# Supplementary material for: A New Spin on Photocatalysis: Vortex Fluidic‐Driven Photooxidations Enabled by Solution‐Processable Microporous Polymers
Source: Chemistry. 2026 Jul 9;32(39):e71288. doi: 10.1002/chem.71288 (PMC13715445; doi:10.1002/chem.71288)
Supplement: Supplementary file 1 [file CHEM-32-e71288-s001.docx]

**Electronic Supporting Information**

**A New Spin on Photocatalysis: Vortex Fluidic Driven Photooxidations Enabled by Solution Processable Microporous Polymers**

Leonardo Amicosante,^a ‡^ Fiona R. Gordon,^a ‡^ Dominic Taylor,^b^ Neil B. McKeown,^b^ Daniel M. Jubb,^c^ Stuart R. Kennedy,^b,c^ Marc A. Little,^a^ Filipe Vilela,^a^ Colin L. Raston,^c^* Scott J. Dalgarno^a^*

^a^Institute of Chemical Sciences, Heriot-Watt University, Riccarton, Edinburgh, Scotland, EH14 4AS, UK.

^b^EaSTCHEM School of Chemistry, University of Edinburgh, Edinburgh, EH9 3FJ, UK.

^c^Flinders Institute of Nanoscale Science and Technology, College of Science and Engineering, Flinders University, Adelaide, SA, 5001 Australia.

^‡^ Authors contributed equally

*Corresponding Authors

Table of Contents

**_______________________________________________________________________________________**

[1.	General Experimental Details	2](#_Toc222900807)

[2.	Synthesis of materials	6](#_Toc222900808)

[2.1.	General Procedure for TB Polymerisation	6](#_Toc222900809)

[3.	Materials characterisation	9](#_Toc222900810)

[4.	VFD Tube coating	21](#_Toc222900811)

[4.1.	Analysis of consistency of the photosensitiser coating	25](#_Toc222900812)

[5.	VFD tests	27](#_Toc222900813)

[5.1.	BTZ loading with respect to starting material	27](#_Toc222900814)

[5.2.	Oxidation of triphenylphosphine	28](#_Toc222900815)

[5.3.	Oxidation of triphenylphosphine with homogeneous photocatalyst	31](#_Toc222900816)

[5.4.	Oxidation of 2-furoic acid	32](#_Toc222900817)

[6.	VFD in continuous flow mode	38](#_Toc222900818)

[6.1.	3D printed photoreactor development	38](#_Toc222900819)

[6.2.	Oxidation of triphenylphosphine in continuous flow mode	39](#_Toc222900820)

[6.3.	Space time yield calculations	42](#_Toc222900821)

[7.	References	44](#_Toc222900822)

# 1. General Experimental Details

^1^H and ^13^C NMR of polymer materials were recorded in CDCl_3_ solution using a 500 MHz Bruker AVIIIHD spectrometer at ambient temperature. Each spectrum was referenced to the residual solvent peak as the internal reference. The **BTZ** content of each polymer was calculated by assuming that peaks in the 7.30-7.80 ppm could be attributed to the **BTZ** group (a total of 8 protons).^1^ Integration against the methyl peaks present in each polymer (a total of 6 protons) gave values of 9.9, 11.1 and 10.1% for **PIM-EA-BTZ-10%**, **P-TOL-BTZ-10%** and **P-DPM-BTZ-10%** respectively. UV-Vis absorption spectra was recorded in CHCl_3_ solution using a PerkinElmer Lambda 25 spectrometer and 10 mm pathlength quartz cuvettes. Infrared (IR) spectra for polymers were recorded using a Shimadzu IRAffinity-1S Fourier Transform Infrared Spectrophotometer on powdered samples.

Gas sorption isotherms were measured for **PIM-EA-BTZ-10%**, **P-TOL-TB**, **P-TOL-BTZ-10%**, **P-DPM-TB** and **P-DPM-BTZ-10%** with a Quantachrome Quandrasorb Evo using N_2_ at 77 K and CO_2_ at 273 K as the adsorbates. Prior to each measurement, the samples were outgassed using a Quantachrome FLOVAC degasser at 100 ^o^C under vacuum for 24 h. Surface areas were calculated from the N_2_ gas adsorption isotherm by applying Brunauer-Emmett-Teller (BET) theory. Pore size distributions for each material were calculated from the CO_2_ gas adsorption isotherm using non-local density functional theory (NLDFT).

The Vortex Fluidic Device used a 20 mm outer diameter (OD) x 17 mm inner diameter (ID) 190 mm length glass quartz tube. Once coated with the PIM-BTZ photosensitiser, the sample tube with suba seal and needle for vent was placed in the VFD. The screw cap and safety shield were secured. A 21V, 600 mA, 410 nm LED lamp was placed on top of the safety shield and secured (Figure S1).

**Figure S1.** VFD setup in confined mode for photo-oxidation reactions. Standard plastic cover with 410 nm lamp and foil (top), and 3D printed reactor with 410 nm lamp rest and internal mirrors for reflectivity (bottom).

The Vortex Fluidic Device in continuous flow mode was setup with the Tricontinent CX6000 syringe pump to add in the reaction mixture and a Vapourtec SF-10 peristaltic pump to pull the product out from the VFD Teflon chamber and collect in a vial.

Tricontinent syringe pump with 6-port valve

Vapourtec SF-10 pump

Vials for reaction mixtures and product collection

410 nm LED light on 3D printed shield

VFD

VFD control box

**Figure S2.** VFD setup in continuous flow mode for photo-oxidation reactions. Standard plastic cover with 410 nm lamp and foil (top), and 3D printed reactor with 410 nm lamp rest and internal mirrors for reflectivity (bottom).

Analysis of the test reactions was carried out using a 400 MHz Bruker AVIIIHD NMR spectrometer at ambient temperature. ^1^H NMR was recorded in D_2_O solution (oxidation of 2-furoic acid) and both ^19^F NMR (hydroxylation of trifluoromethylphenylboronic acid) and ^31^P NMR (oxidation of triphenylphosphine) were recorded in CDCl_3_ solution. Analysis of phosphine oxidation reactions used an inverse gated sequence, a D1 of 40 s and an acquisition time of 2.5 seconds. Diphenylphosphine oxide (DPPO) was used as an internal standard to confirm the inverse gated ^31^P NMR experiment was quantitative. For the oxidation of triphenylphosphine, the starting reaction mixture (0.2 mL) was sampled along with 0.05 mmol of DPPO in CDCl_3_ and resulting spectra compared to the spectra of the product reaction mixture (0.2 mL) with 0.05 mmol DPPO in CDCl_3_. Results proved to show that the ^31^P NMR experiment was quantitative and a suitable method to validate the test experiments carried out. The integration of product and reactant peaks in the resulting spectra were used to calculate the percentage conversion for each test reaction.

# 2. Synthesis of materials

**DPM** and **TOL** were both commercially available and used without further purification. **EA** and **PIM-EA-TB** were prepared as described by Carta *et al.* ^2^**pNH_2_-BTZ** and all TB polymers were prepared by modifying literature procedures reported by Taylor *et al*.^1^

## 2.1. General Procedure for TB Polymerisation

Diamine (**EA** or **TOL** or **DPM**) (3.15 or 3.50 mmol) and **pNH_2_-BTZ** (0.35 or 0 mmol) were dissolved in dimethoxymethane (DMM) (1.6 mL, 17.85 mmol) and the solution cooled using an ice-water bath. Under a nitrogen atmosphere, trifluoroacetic acid (TFA) (8 mL) was added drop-wise over 30 minutes, then the mixture was stirred for 48 hours at room temperature. The solution was next poured onto a mixture of 28% aqueous ammonia (10 mL) in ice water (100 mL) and stirred for 2 hours. The precipitate was collected by filtration, washed with water and acetone until the washings ran clear. The crude polymer was then purified twice by reprecipitation of chloroform solution (2 x 35 mL) into *n*-hexane (2 x 200 mL). Finally, the precipitated powder was refluxed in methanol for 48 h, filtered and dried in a vacuum oven at 100 ^o^C for 6 h.

**PIM-EA-BTZ-10%**

**PIM-EA-BTZ-10%** was prepared using the general TB polymerisation procedure using **EA** (833 mg, 3.15 mmol) and **pNH_2_-BTZ** (111 mg, 0.35 mmol) to yield the product as a light-yellow powder (658 mg, 73% recovery).

**P-TOL-TB**

**P-TOL-TB** was prepared using the general TB polymerisation procedure using **TOL** (743 mg, 3.5 mmol) to yield the product as a white powder (662 mg, 76% recovery).

**P-TOL-BTZ-10%**

**P-TOL-BTZ-10%** was prepared using the general TB polymerisation procedure using **TOL** (833 mg, 3.15 mmol) and **pNH_2_-BTZ** (111 mg, 0.35 mmol) to yield the product as a light-yellow powder (658 mg, 73% recovery).

**P-DPM-TB**

**P-DPM-TB** was prepared using the general TB polymerisation procedure using **DPM** (792 mg, 3.5 mmol) yield the product as a white powder (606 mg, 66%).

**P-DPM-BTZ-10%**

**P-DPM-BTZ-10%** was prepared using the general TB polymerisation procedure using **DPM** (712 mg, 3.15 mmol) and **pNH_2_-BTZ** (111 mg, 0.35 mmol) to yield the product as a light-yellow powder (769, 81%).

# 3. Materials characterisation

**Figure S3.**^1^H NMR spectra of **PIM-EA-BTZ-10%** in CDCl_3_.

**Figure S4.** ^13^C NMR spectra of **PIM-EA-BTZ-10%** in CDCl_3_.

**Figure S5.**^1^H NMR spectra of **P-TOL-TB** in CDCl_3_.

**Figure S6.** ^13^C NMR spectra of **P-TOL-TB** in CDCl_3_.

**Figure S7.** ^1^H NMR spectra of **P-TOL-BTZ-10%** in CDCl_3_.

**Figure S8.** ^13^C NMR spectra of **P-TOL-BTZ-10%** in CDCl_3_.

**Figure S9.** ^1^H NMR spectra of **P-DPM-TB** in CDCl_3_.

**Figure S10.** ^13^C NMR spectra of **P- DPM-TB** in CDCl_3_.

**Figure S11.** ^1^H NMR spectra of **P- DPM-BTZ-10%** in CDCl_3_.

**Figure S12.** ^13^C NMR spectra of **P-DPM-BTZ-10%** in CDCl_3_.

**Figure S13.** Normalised UV-Vis absorption spectra for **PIM-EA-TB** (grey) and **PIM-EA-BTZ-10%** (blue) in CHCl_3_ solution.

**Figure S14.** Normalised UV-Vis absorption spectra for **P-TOL-TB** (grey) and **P-TOL-BTZ** (blue) in CHCl_3_ solution.

**Figure S15.** Normalised UV-Vis absorption spectra for **P-DPM-TB** (grey) and **P-DPM-BTZ** (blue) in CHCl_3_ solution.

**Figure S16.** IR spectra of TB polymers studied.

**Figure S17.** N^­^_2_ gas adsorption (filled) and desorption (hollow) isotherm at 77 K for **PIM-EA-BTZ-10%** (blue triangles).

**Figure S18.** N^­^_2_ gas adsorption (filled) and desorption (hollow) isotherms at 77 K for **P-TOL-TB** (grey circles) and **P-TOL-BTZ-10%** (blue triangles).

**Figure S19.** N^­^_2_ gas adsorption (filled) and desorption (hollow) isotherms at 77 K for **P-TOL-TB** (grey circles) and **P-TOL-BTZ-10%** (blue triangles).

**Figure S20.** CO^­^_2_ gas adsorption isotherms at 273 K for **PIM-EA-BTZ-10%** (red filled triangles), **P-TOL-TB** (yellow hollow squares), **P-TOL-BTZ-10%** (yellow filled squares), **P-DPM-TB** (blue hollow diamonds) and **P-DPM-BTZ-10%** (blue filled diamonds).

**Figure S21.** Pore size distribution for **PIM-EA-BTZ-10%**.

**Figure S22.** Pore size distribution for **P-TOL-TB**.

**Figure S23.** Pore size distribution for **P-TOL-BTZ-10%**.

**Figure S24.** Pore size distribution for **P-DPM-TB**.

**Figure S25.** Pore size distribution for **P-DPM-BTZ-10%**.

# 4. VFD Tube coating

Slow rotation evaporation coating method: **PIM-EA-BTZ-10%** (20 mg) dissolved in dichloromethane (1 mL) was added to the VFD tube. The VFD was setup in the confined mode with 0° tilt angle and was rotated at 500 rpm to allow for slow evaporation. After 24 hours the DCM had fully evaporated, and the resulting film appeared as a striped coating (Figure S26).

**Figure S26.** **PIM-EA-BTZ-10%** coating attempt at a 0° tilt angle with slow evaporation of DCM.

Fast rotation coating method: **PIM-EA-BTZ-10%** (53 mg) was dissolved in DCM (2 mL) and added to the VFD tube. In the confined mode the tube was rotated at 6000 rpm with a 45° tilt angle. The film began to form after 1 hour and a heat gun was used to evaporate the remaining DCM with the resulting film appearing thick and patchy (Figure S27).

**Figure S27.** **PIM-EA-BTZ-10%** coating attempt at a 45° tilt angle with heat gun for quick evaporation of DCM.

Gentle evaporation coating method: **PIM-EA-BTZ-10%** (20 mg) was dissolved in DCM (2 mL) and added to the VFD tube. In the confined mode the tube was rotated at 500 rpm with a 0° tilt angle. A heat gun was used to gently evaporate the DCM however, this resulted in patches of coating which was not ideal (Figure S28).

**Figure S28.** **PIM-EA-BTZ-10%** coating attempt at a 0° tilt angle with heat gun for gentle evaporation of DCM.

Rotary evaporator coating method: **P-TOL-BTZ-10%** (2 mg) was dissolved in chloroform (0.5 mL) and added to the VFD tube. The rotary evaporator method was used to remove the solvent and slowly coat the tube (Figure S29). More control was gained by using the rotary evaporator.

**Figure S29.** Rotary evaporator method to create thin film coating of the PIM-BTZ photosensitiser in the VFD tube.

**Table S1.** Photosensitiser (PS) **P-TOL-BTZ-10%** coating formed using rotary evaporator method and durability investigated over a 24 hour period while rotating solvent. Pictures show before and after the 24 hours experiment. Results show that deionised water did disrupt the film appearance and cause delamination however with methanol very little to no delamination was observed.

| Entry | Mass of PS / mg | Rotation speed / rpm | Solvent | PS coating before experiment | PS coating after 24 hour experiment |
| --- | --- | --- | --- | --- | --- |
| 1 | 2 | 2000 | Deionised water |  |  |
| 2 | 2 | 6000 | Deionised water |  |  |
| 3 | 10 | 6000 | Deionised water |  |  |
| 4 | 2 | 3000 | Methanol |  |  |

VFD injection coating method: The sample tube was placed in the VFD, tilt angle set to 45° and rotation speed set to 6000 rpm. In a vial, a concentrated solution of **P-TOL-BTZ-10%** (2 mg) in DCM (0.4 mL) for coating the VFD tube was made. With the VFD spinning, the coating solution was slowly injected into the bottom of the VFD tube whilst carefully moving the needle upwards. This gave a consistent coating, and this method was continued to be used to coat the VFD tubes with the PIM photosensitisers for the remainder of the study.

**Table S2.** Investigation of the durability of the **P-TOL-BTZ-10%** film coating, made via the VFD high rotation inject method, after 24 hours of spinning methanol and deionised water at 6000 rpm.

| Solvent | Time spinning / h | Photosensitiser Coating |
| --- | --- | --- |
| Methanol | 0 (before spinning) |  |
| Methanol | 24 |  |
| Methanol | After rinsing with methanol |  |
| Deionised Water | 0 (before spinning) |  |
| Deionised Water | 24 |  |
| Deionised Water | After rinsing with methanol |  |

## 4.1. Analysis of consistency of the photosensitiser coating

For the reusability of the **P-TOL-BTZ-10%** photosensitiser coated tubes, the coating coverage was analysed using the Thorlabs PM100D optical power/energy meter (Figure S30). The results confirmed that the coating remained with minor delamination between experiments shown by a slight decrease of optical penetration observed by the detector (Table S3). This lower value may seem unexpected but as the coating delaminates it becomes distorted meaning less light may hit the detector when measuring at the same distance.

**Figure S30.** The Thorlabs PM100D optical power/energy meter used for monitoring the coverage of the photosensitiser coating of each tube.

**Table S3.** Analysis of the photosensitiser coating using the Thorlabs PM100D optical power/energy meter to measure the light penetration after each reusability experiment of the same coated tube.

| Reusability of tube exp | Detected optical power /mW | | | |
| --- | --- | --- | --- | --- |
|  | Top | Middle | Bottom | Average |
| 0 (pre-experiments) | 1.94 | 1.90 | 1.96 | 1.93 |
| 1 | 1.90 | 1.81 | 1.81 | 1.84 |
| 2 | 1.84 | 1.81 | 1.79 | 1.81 |
| 3 | 1.80 | 1.82 | 1.67 | 1.76 |
| 4 | 1.78 | 1.70 | 1.61 | 1.70 |
| 5 | 1.75 | 1.70 | 1.60 | 1.68 |

Three tubes coated in the P-TOL-BTZ-10% photosensitiser *via* the VFD high rotation coating method were also analysed using the Thorlabs PM100D optical power/energy meter (Figure S30). This was used to quantify how much light penetrates through the coating. The results confirmed that for each coating similar coverage was observed. (Table S4).

**Table S4.** Analysis of the photosensitiser coverage using the Thorlabs PM100D optical power/energy meter to measure the light penetration of three coated tubes.

| Tube no. | Detected optical power /mW | | | |
| --- | --- | --- | --- | --- |
|  | Top | Middle | Bottom | Average |
| 1 | 1.91 | 1.86 | 1.86 | 1.88 |
| 2 | 1.84 | 1.84 | 1.88 | 1.85 |
| 3 | 1.94 | 1.90 | 1.96 | 1.93 |

# 5. VFD tests

## 5.1. BTZ loading with respect to starting material

For each test 0.05 mmol of the starting material was used with the **P-TOL-BTZ-10%** (2 mg) coated tube. The percentage **BTZ** loading in relation to reactant was calculated as follows:

$$Molecular weight of P-TOL=278.40 mg/mmol$$

$$Molecular weight of BTZ=354.43 mg/mmol$$

Thus, the combined molecular weight can be obtained by factoring in the partial ratio of each monomer as follows:

$$\text{P-TOL-BTZ-10\%} monomer weight=\left( 278.40\times0.9 \right)+\left( 354.4\times0.1 \right)$$

$$\text{ }=285.64 mg/mmol$$

$$total mmol=\frac{2 mg}{285.64 mg/mmol}=0.007 mmol$$

$$10\% mmol=0.0007 mmol$$

Therefore, the total loading with respect to reactant quantity (0.05 mmol) can be calculated:

$$\text{BTZ loading }=\frac{0.0007mmol}{0.05mmol}\times100=\boldsymbol{1.4\% BTZ loading}$$

Eq. S1

## 5.2. Oxidation of triphenylphosphine

In a **P-TOL-BTZ-10%** coated VFD tube, triphenylphosphine (0.05 mmol, 13 mg) was added and dissolved in methanol (2 mL). A suba seal with small needle was added to the tube which was placed in the VFD at a 45° tilt angle with screw cap and safety shield on. The 410 nm light was attached to the safety shield and switched on. The rotation speed was set to 6000 rpm and the reaction proceeded for 40 min.

After 40 min, the VFD and light were switched off. An NMR sample was taken (0.2 mL reaction mixture in 0.5 mL CDCl_3_) and quantitative ^31^P NMR experiment conducted, resulting in 100% conversion. Methanol was removed under reduced pressure to yield the product. Resulting NMR spectra agreed with previously published literature.^3^

Conversion / %

| Coating Reusability Run | Conversion / % |
| --- | --- |
| 1 | 80 |
| 2 | 89 |
| 3 | 89 |
| 4 | 88 |
| 5 | 82 |

**Figure S31.** Percentage conversion from the reusability of the **P-TOL-BTZ-10%** coated VFD tube for the oxidation of triphenylphosphine.

| A) Delamination after 4^th^ reusability experiment |  |
| --- | --- |
| B) Delamination after 5^th^ reusability experiment |  |

**Figure S32.** Post experiment rinsing of the photosensitiser coated tube using methanol for 4^th^ and 5^th^ reusability of coating experiments. Washings examined under UV light and showed some delamination. A) Some delamination in washings after the 4^th^ reusability experiment and B) small quantities of delamination in washings after the 5^th^ reusability experiment.

Conversion / %

| Coating Reproducibility Number | Conversion / % |
| --- | --- |
| 1 | 78 |
| 2 | 80 |
| 3 | 82 |

**Figure S33.** Reproducibility of the **P-TOL-BTZ-10%** photosensitiser coating for the oxidation of triphenylphosphine. Experiments carried out over 30 min at 2000 rpm with 45° tilt angle.

| A) First reproducibility |  |
| --- | --- |
| B) Second reproducibility |  |
| C) Third reproducibility |  |

**Figure S34.** Appearance of P-TOL-BTZ-10% coated VFD tubes for each of the reproducibility experiments.

**Table S5.** Molar equivalents of singlet oxygen quencher, DABCO, required to trap singlet oxygen and stop the oxidation of triphenylphosphine in batch vs VFD.

| Reactor | Molar Equivalents of DABCO | Conversion / % |
| --- | --- | --- |
| Batch | 10 | 0 |
| VFD (photosensitiser free in solution) | 10 | 0 |
| VFD | 2 | 56 |
| VFD | 10 | 50 |
| VFD | 26 | 18 |
| VFD | 64 | 0 |

The temperature of the VFD tube was monitored using an IR temperature sensor over the 30 min reaction time for the oxidation of triphenylphosphine. Only a small increase in temperature was observed (Table S6).

**Table S6.** Temperature observation of the VFD tube during the oxidation of triphenylphosphine over 30 min. Image shows sections of the tube which had temperature recorded.

| Reaction time / min | Temperature / °C | | | |
| --- | --- | --- | --- | --- |
|  | **Through** | **Top** | **Middle** | **Bottom** |
| 0 | 22.7 | 22.9 | 22.9 | 22.7 |
| 15 | 24 | 23.6 | 23.6 | 23.5 |
| 30 | 25.2 | 23.5 | 23.5 | 23.5 |

## 5.3. Oxidation of triphenylphosphine with homogeneous photocatalyst

In a VFD tube, **P-TOL-BTZ-10%** (2 mg), triphenylphosphine (0.05 mmol, 13 mg) was added and dissolved in chloroform (2 mL). A suba seal with small needle was added to the tube which was placed in the VFD at a 45° tilt angle with screw cap and safety shield on. The 410 nm light was attached to the safety shield and switched on. The rotation speed was set to 6000 rpm and the reaction proceeded for 10 min.

After 30 min, the VFD and light were switched off. An NMR sample was taken (0.2 mL reaction mixture in 0.5 mL CDCl_3_) and quantitative ^31^P NMR experiment conducted, resulting in 100% conversion. Chloroform was removed under reduced pressure to yield the product. Resulting NMR spectra agreed with previously published literature.^3^

**Table S7.** Comparison of homogenous **P-TOL-BTZ-10%** photosensitiser vs heterogeneous.

| Factor | Homogeneous Photosensitiser | Heterogeneous Photosensitiser |
| --- | --- | --- |
| Reaction solvent | Chloroform or DCM | Methanol or water |
| Recovery of photosensitiser | None | Coated the tube so can be recovered by dissolving in min volume of DCM/chloroform then isolated. |
| Use of problematic chlorinated solvents | 2 mL for reaction solvent for each exp | 0.4 mL to make coating but used for five consecutive exps, minimal volume to recover photosensitiser. |

## 5.4. Oxidation of 2-furoic acid

To a **P-TOL-BTZ-10%** coated VFD tube, 2-furoic acid (0.05 mmol, 6 mg) was added and dissolved in deionised water (2 mL). A suba seal with small needle was added to the tube and the tube placed in the VFD at a 45° tilt angle with screw cap and safety shield on. The 410 nm light was attached to the safety shield with aluminium foil used to shield light from the chemist’s eyes. The rotation speed was set to 6000 rpm, 410 nm light on and the reaction proceeded for 24 h. After 24 h, the VFD and light were switched off. A sample was taken and analysed *via* ^1^H NMR, resulting in 100% conversion. The product was not isolated, and crude analysis was agreed with literature values.^4^

**5.4.	Hydroxylation of 4-trifluoromethylphenylboronic acid**

To a P-TOL-BTZ-10% coated VFD tube, 4-trifluoromethylphenylboronic acid (0.05 mmol, 9.5 mg) was added and dissolved in methanol (2 mL). A suba seal with small needle was added to the tube which was placed in the VFD at a 45 ° tilt angle with screw cap and safety shield on. The 410 nm light was attached to the safety shield and switched on. The rotation speed was set to 7500 rpm and the reaction proceeded for 4 h. After 4 h, the VFD and light were switched off. A sample was taken and analysed via ^19^F NMR, resulting in 100% conversion. The solvent was removed under reduced pressure to yield the product. Resulting NMR spectra agreed with previously published literature.^3^

**Figure S35.** ^19^F NMR of the trifluoromethylphenylboronic acid starting material (‑63.4 ppm) in CDCl_3_.

**Figure S36.** ^19^F NMR of product, 4-trifluoromethylphenol (-61.9 ppm) in CDCl_3_.

**Table S8.** Optimisation of the hydroxylation of 4-(trifluoromethyl)phenyl boronic acid to 4-(trifluoromethyl) phenol in the VFD via O_2_^•-^.

| Entry | Photosensitiser | PS Loading / mg | Tilt angle / ° | Rotation speed / rpm | Time / h | Conversion / % |
| --- | --- | --- | --- | --- | --- | --- |
| 1 | **P-TOL-BTZ-10%** | 2 | 0 (horizontal) | 6000 | 2 | **17** |
| 2 | **P-TOL-BTZ-10%** | 2 | 30 | 6000 | 2 | **28** |
| 3 | **P-TOL-BTZ-10%** | 2 | 45 | 6000 | 2 | **55** |
| 4 | **P-TOL-BTZ-10%** | 2 | 60 | 6000 | 2 | **31** |
| 5 | **P-TOL-BTZ-10%** | 2 | 90 (vertical) | 6000 | 2 | **25** |
| 6 | **P-TOL-BTZ-10%** | 2 | 45 | 2000 | 2 | **34** |
| 7 | **P-TOL-BTZ-10%** | 2 | 45 | 3000 | 2 | **28** |
| 8 | **P-TOL-BTZ-10%** | 2 | 45 | 4500 | 2 | **47** |
| 9 | **P-TOL-BTZ-10%** | 2 | 45 | 7500 | 2 | **63** |
| 10 | **P-TOL-BTZ-10%** | 2 | 45 | 9000 | 2 | **21** |
| 11 | **P-TOL-TB** | 2 | 45 | 6000 | 2 | **3** |
| 12 | **PIM-EA-BTZ-10%** | 2.7 | 45 | 6000 | 2 | **18** |
| 13 | **PIM-EA-TB** | 2.7 | 45 | 6000 | 2 | **2** |
| 14 | **P-DPM-BTZ-10%** | 2.1 | 45 | 6000 | 2 | **8** |
| 15 | **P-DPM-TB** | 2.1 | 45 | 6000 | 2 | **0** |

**Table S9.** Molar equivalents of TEMPO and DABCO vs the conversion of the boronic acid hydroxylation. Each reaction carried out over 2 h, using 0.05 mmol boronic acid starting material, VFD rotation speed = 6000 rpm, tilt angle 45°.

| Reactor | Molar Equivalents of TEMPO | Molar Equivalents of DABCO | Conversion / % |
| --- | --- | --- | --- |
| Batch | 10 | 0 | **0** |
| Batch | 64 | 0 | 0 |
| No needle VFD | 10 | 0 | 28 |
| No needle VFD | 64 | 0 | 0 |
| VFD | 2 | 0 | 26 |
| VFD | 10 | 0 | 26 |
| VFD | 30 | 0 | 24 |
| VFD | 64 | 0 | 20 |
| VFD | 0 | 30 | 23 |
| VFD | 64 | 64 | 0 |

To verify that the reaction proceeded *via* the superoxide radical, control tests with a quencher were carried out using TEMPO. To be consistent with the DABCO quenching of singlet oxygen, 64 molar equivalents were used as the previous trapping tests verified that any lesser amount did not guarantee full reaction quenching (whereas batch trapping tests often rely on 2 molar equivalents). However, it was observed that 64 equivalents of both TEMPO and DABCO were required to completely halt the reaction (Table S7). The confined mode VFD setup of previous experiments included a needle through the suba seal acting as an aerobic vent. To gain a better understanding of the volume of reactive oxygen species being formed, the reaction was carried out using no needle through the capped seal, preventing oxygen from continuously entering the vessel. As a consequence, 64 equivalents of TEMPO alone stopped the reaction. This emphasises that a great amount of oxygen is entering through the needle, and some may be converted to singlet oxygen or other oxygen species, which contribute to the product through a separate mechanism. Additional modelling is to be conducted in future studies to thoroughly understand this effect. However, the test in confined aerobic conditions provides proof that the superoxide radical is required for the reaction to proceed, and TEMPO traps the radical produced by the photocatalyst polymer.

# 6. VFD in continuous flow mode

## 6.1. 3D printed photoreactor development

The new shield/rest for the 410 nm LED lamp was designed using Autodesk Inventor and sliced using the Anycubic Photon Workshop software, to be loaded and printed using a resin Photon Mono X printer. Due to the printer size limitations, the shield was divided in 3 separate parts to be then built up and collated, adding to each side commercially available reflective mirrors, granting internal reflectivity and overall stability to the structure.

**Figure S37.** Light shield had to be printed in two halves. Left = 3D printing of light shield. Right = 3D printed half being cured.

**Figure S38.** Final light shield with halves superglued together and acrylic mirrors attached inside to reflect light.

## 6.2. Oxidation of triphenylphosphine in continuous flow mode

A **P-TOL-BTZ-10%** (2 mg) coated sample tube was placed in the VFD and the reaction mixture in a separate vial contained triphenylphosphine (0.5 mmol, 130 mg) dissolved in methanol (20 mL). The syringe pump was setup with one tube pulling from the reaction mixture vial to dispense through a needle into the VFD. A 1 mL syringe was used with a flow rate of 0.1 mL/min, giving a residence time of 18 min. The rotation speed was set to 7500 rpm, tilt angle at 45° with 410 nm light. Once setup was confirmed the experiment commenced. As the reaction mixture rotated in the VFD tube, it moved up the tube *via* the centrifugal forces and exited using the peristaltic pump to guide the mixture to the product collection vial. An NMR sample was taken from this vial (0.2 mL product mixture in 0.5 mL CDCl_3_) and quantitative ^31^P NMR experiment conducted, resulting in 100% conversion.

**Table S10.** Triphenylphosphine oxidation under continuous flow conditions in the VFD with **P-TOL-BTZ-10%** photosensitiser coated tube.

| Reaction mixture flow rate / mL min ^-1^ | Residence time / min | Reaction time / h | Tilt angle /° | Rotation speed / rpm | Conversion / % |
| --- | --- | --- | --- | --- | --- |
| 0.2 | 9 | 0.30 | 45 | 7500 | 50 |
| 0.1 | 18 | 0.67 | 45 | 7500 | 100 |
| 0.1 | 18 | 3.33 | 45 | 7500 | 100 |
| 0.1 | 18 | 3.33 | 45 | 6000 | 100 |
| 0.2^*^ | 9 | 1.67 | 45 | 6000 | 100 |
| 0.3^*^ | 6 | 1.11 | 45 | 6000 | 100 |
| 0.4^*^ | 4.5 | 0.83 | 45 | 6000 | 82 |

^*^New 3D printed light shield used

**Table S11.** Comparison of reactors for the oxidation of triphenylphosphine.

| Reactor | Batch  (vial) | VFD  (no spin) | Confined mode VFD | Continuous flow VFD | Continuous flow VFD (3d printed shield) |
| --- | --- | --- | --- | --- | --- |
| Photosensitiser | **P-TOL-BTZ-10%** | **P-TOL-BTZ-10%** | **P-TOL-BTZ-10%** | **P-TOL-BTZ-10%** | **P-TOL-BTZ-10%** |
| Photosensitiser functionality | Heterogeneous suspension | Coated VFD tube | Coated VFD tube | Coated VFD tube | Coated VFD tube |
| Residence Volume / mL | 2 | 2 | 2 | 1.8 | 1.8 |
| Reactor Volume / mL | 22 | 40 | 40 | 40 | 40 |
| Reaction Time / h | 0.5 | 0.5 | 0.5 | 3.33 | 1.11 |
| Product made / mg | 7.5 | 5.3 | 13.1 | 139 | 139 |
| Space time yield / mg h^-1^ mL^-1^ (residence volume) | **7.5** | **5.3** | **13.1** | **23.2** | **70.2** |
| Space time yield / mg h^-1^ mL^-1^ (reactor volume) | **0.681** | **0.265** | **0.655** | **1.044** | **3.131** |
| Productivity / mg h^-1^ | **15.0** | **10.6** | **26.2** | **41.7** | **126.4** |

## 6.3. Space time yield calculations

The value of space time yield (STY) was calculated as follows for the continuous flow mode VFD (Figure 5 in the main manuscript) using the values below:^5,6^

Reactor Volume (40 mL); Residence Volume (1.8 mL); Flow rate pump A (0.1 mL min^-1^); Reaction time (3.33 hours); Residence time (18 min, 0.3 hours); Yield (full conversion) (139 mg).

The residence volume (and therefore the maximum volume present in the reactor when equilibrium is reached) was obtained as the product of the measured residence time and the flow rate of pump A (inlet) (Figure 5 in main manuscript).

$$Residence Volume=Residence time \left( 18 min \right) x Flow rate of Pump A \left( 0.1\frac{\mathrm{mL}}{\min} \right)=1.8 mL$$

Eq. S2

The space time yield (STY) of the system can be obtained as follows:^5^

$$STY=\frac{\mathrm{Yield}\left( \mathrm{mg} \right)}{Reaction Time \left( h \right) x Residence Volume \left( \mathrm{mL} \right)}$$

Eq. S3

Thus, input of experimental variables of the continuous flow VFD system give:

$$STY (VFD)=\frac{139 mg}{3.33 h x 1.8 mL}= 23.2 mg h^{-1}\mathrm{mL}^{-1}$$

Eq. S4

The same calculation can be run for batch test:

$$STY (vial)=\frac{7.5 mg}{0.5 h x 2 mL}= 7.5 mg h^{-1}\mathrm{mL}^{-1}$$

Eq. S5

And for reference the space-time yield of literature can be obtained the same way:^1^

$$STY (column reactor)=\frac{51.8 mg}{18 h x 1.9 mL}= 1.5 mg h^{-1}\mathrm{mL}^{-1}$$

Eq. S6

The space time yield (STY) using reactor volume can be calculated as follows:

$$STY=\frac{\mathrm{Yield}\left( \mathrm{mg} \right)}{Reaction Time \left( h \right) x Reactor Volume \left( \mathrm{mL} \right)}$$

Eq. S7

For the continuous flow VFD system this gives:

$$STY (VFD)=\frac{139 mg}{3.33 h x 40 mL}= 0.655 mg h^{-1}\mathrm{mL}^{-1}$$

Eq. S8

The productivity of the system can be calculated as follows:

$$Productivity= \frac{Yield (mg)}{Reaction Time (h)}$$

Eq. S9

Thus, input of experimental variables of the continuous flow VFD system give:

$$\mathrm{Productivity}\left( \mathrm{VFD} \right)= \frac{139 mg}{3.33 h}=41.7 mg h^{-1}$$

Eq. S10

For batch experimentation values it gives:

$$\mathrm{Productivity}\left( \mathrm{Batch} \right)= \frac{7.5 mg}{0.5 h}=15.0 mg h^{-1}$$

Eq. S11

# 7. References

1	D. Taylor, J. M. Tobin, L. Amicosante, A. W. Prentice, M. J. Paterson, S. J. Dalgarno, N. B. McKeown and F. Vilela, Immobilisation of benzo[c][1,2,5]thiadiazole (BTZ) within polymers of intrinsic microporosity (PIMs) for use in flow photochemistry, *J. Mater. Chem. A*, 2024, **12**, 10932–10941.

2	M. Carta, R. Malpass-Evans, M. Croad, Y. Rogan, J. C. Jansen, P. Bernardo, F. Bazzarelli and N. B. McKeown, An Efficient Polymer Molecular Sieve for Membrane Gas Separations, *Science (80-. ).*, 2013, **339**, 303–307.

3	J. M. Tobin, T. J. D. McCabe, A. W. Prentice, S. Holzer, G. O. Lloyd, M. J. Paterson, V. Arrighi, P. A. G. Cormack and F. Vilela, Polymer-Supported Photosensitizers for Oxidative Organic Transformations in Flow and under Visible Light Irradiation, *ACS Catal.*, 2017, **7**, 4602–4612.

4	H. Urakami, K. Zhang and F. Vilela, Modification of conjugated microporous poly-benzothiadiazole for photosensitized singlet oxygen generation in water, *Chem. Commun.*, 2013, **49**, 2353–2355.

5	A Practical Guide to Catalyst Testing, Catalytica, 1987.

6	D. S. Lee, Z. Amara, C. A. Clark, Z. Xu, B. Kakimpa, H. P. Morvan, S. J. Pickering, M. Poliakoff and M. W. George, Continuous Photo-Oxidation in a Vortex Reactor: Efficient Operations Using Air Drawn from the Laboratory, *Org. Process Res. Dev.*, 2017, **21**, 1042–1050.
